# Large scale clustering of AlphaFold2 3D models shines light on the structure and function of proteins

**DOI:** 10.1016/j.molcel.2023.10.039

**Published:** 2023-11-16

**Authors:** Nicola Bordin, Andy M. Lau, Christine Orengo

**Affiliations:** 1 Institute of Structural and Molecular Biology, University College London, WC1E 6BT, London, UK; 2 Department of Computer Science, University College London, WC1E 6BT, London, UK

## Abstract

Two recent studies exploited ultra-fast structural aligners and deep-learning approaches to cluster the protein structure space in the AlphaFold Database. Barrio-Hernandez et al. (2023)^2^ and Durairaj et al. (2023)^3^ uncovered fascinating new protein functions and structural features previously unknown.

Proteins drive the machinery of cells and viruses, regulating their replication processes and interactions with the environment. Over billions of years, their shapes in 3D space evolved to perform different roles across the tree of life, tightly linking structures to their functions. Structure can be inferred from a protein’s sequence, with the latter data expanding massively with the recent improvements in sequencing technologies and metagenome initiatives. Structural data also grew exponentially but much more slowly, with nearly 300-fold more sequences recently. This was due to the difficulty and time involved with experimental approaches and the need for close structural homologues to generate 3D-models. Borrowing a term from astronomy, the protein sequence universe used to be a dark space, with a few structures illuminating its functions and shapes (Figure 1).

Recent advances in deep-learning-based methods to predict structure from sequence (e.g., AlphaFold2) have bridged this gap significantly with models that are often highly accurate^1^. The AlphaFold Database, containing over 200 million models, has brightened a large swath of the protein universe with structural information. This universe is a vast space to traverse using traditional search methods, but the emergence of ultra-fast sequence and structural aligners (such as MMseqs2 and Foldseek) enabled two recent studies by Barrio-Hernandez et al.^2^ and Durairaj et al.^3^ to cluster the AlphaFold protein structure space, allowing the discovery of novel functions and previously unseen 3D-shapes.

Alongside the steady contribution of individual experimental structures in the Protein Data Bank (PDB), many large-scale attempts have been made to characterise the protein universe since its first definition in 1992 as the collection of all proteins in all organisms. For example, the Protein Structural Genomics Initiative (PSI) focused structure determination on under-sampled regions of sequence space, targeting very large families of proteins with low structural coverage^4^. PSI contributed over 8% of all deposited protein structures in the PDB in a 5-year period, but experimental techniques are very time consuming. As with a traditional rocket ship, experimental techniques won’t be able to reach the edge of the visible Protein Universe, whereas next-generation sequencing could be considered to travel at the speed of light.

Theoretically, there are over 20^100^ possible amino acid combinations in a protein with 100 residues, though almost all of these combinations are not foldable into a 3D structure^5^. Therefore, many past approaches identified domain structure folds (globular constituents of proteins) in the experimental data (PDB). These fold ‘building blocks’ were mapped onto constellations (sequence families, e.g., in Pfam) and galaxies in the protein universe using sequence profiles linked to structural families (e.g., in SCOP, CATH) enabling extensive exploration of the ways in which folds had been combined^6,7^. However, a deeper understanding of functions in the protein universe required precise 3D-shapes.

Whilst there have been steady improvements in sequence and structure-based tools for clustering the protein universe, recent astonishing advances in artificial intelligence (AI)-based methods have provided the most powerful torch to structurally light the darkness. Building on the shoulders of the Protein Data Bank, and exploiting the vast sequence data available, AlphaFold and recent predictors leveraged the wealth of information encoded in these sequences and structures. This AI model predicts 3D-models with stunning accuracy often comparable to experimental methods, although some islands with low sequence population or high levels of disorder may be less tractable^8^. Targeting UniProt, the AlphaFold Database (AFDB) grew to cover almost its entirety with over 200 million models available as of 2023. Suddenly, the protein structure space is bright with light, enabling research that was previously hindered by the lack of protein structure.

Initial community efforts exploited AlphaFold data for 21 model organisms to group similar 3D-shapes into clusters, detect novel folds and putative interactions, reveal levels of disorder and highlight the benefits for medicine and basic research^9^. However, tools built pre-AlphaFold did not scale well to the sheer deluge of 200 million structural models in AFDB. Serendipitously, AlphaFold2 coincided with the first release of Foldseek, an extremely fast structural aligner developed by the Steinegger group.

In their recent work the Steinegger group and collaborators clustered AFDB into just 2.3 million clusters using Foldseek. This monumental task which would have taken 10 years on a 64-core computer prior to Foldseek was accomplished in 5 days with comparable accuracy^2^. Most of the clusters identified (69%) resemble structures previously seen in the PDB and contain proteins from organisms distributed across the Tree of Life, whilst some clusters have species-specific novelties, suggesting a potential ‘evolutionary sandbox’ where novel functions can emerge. Remarkably, proteins with putative immune-related roles in humans were clustered with ancient prokaryotic proteins, revealing fascinating evolutionary trajectories. Using novel deep-learning methods (e.g., DeepFri^10^) they annotated proteins lacking experimental functions. Many of these are related to “transport” and “transmembrane” functions, which are elusive in the PDB due to their difficulty in structural determination.

Durairaj and colleagues gave an alternative view of the protein universe by first assessing the available functional annotations for UniProt proteins clustered together at 50% sequence identity (UniRef50). They reported ‘functional brightness’, identifying 34% of UniRef50 clusters (containing >30 million proteins) devoid of any prior clues regarding function. Leveraging the AFDB coverage for 78% of UniRef50 clusters, they used MMseqs to link clusters annotated with high quality models in order to inherit functions from structures in close proximity. This illuminated over 40% of the dark clusters. Deeper analyses of some previously unannotated UniRef50 clusters now connected in the MMseqs network, using DeepFri and other approaches, revealed a glycosyltransferase family and a previously unknown toxin-antitoxin system that was validated experimentally. A novel AI strategy based on structural embeddings compared the AFDB structures to the PDB data identifying roughly 700,000 structural outliers in fold space, including a beautiful new β-flower fold.

The AlphaFold Database vastly expanded our knowledge of fold space, but there are still regions (known as the ‘dark proteome’) where poor quality models, intrinsically disordered proteins, viral proteins (currently not available in AFDB) and structural novelty in metagenomes remain as ‘black holes’ within the protein universe. Since disordered regions can contain important clues to function, these recent large-scale approaches help identify areas where experimental efforts could be focused to address localised areas of darkness. Many new biological questions can be posed now that we have such a vast new protein structure universe to explore.

## Figures and Tables

**Figure 1:**
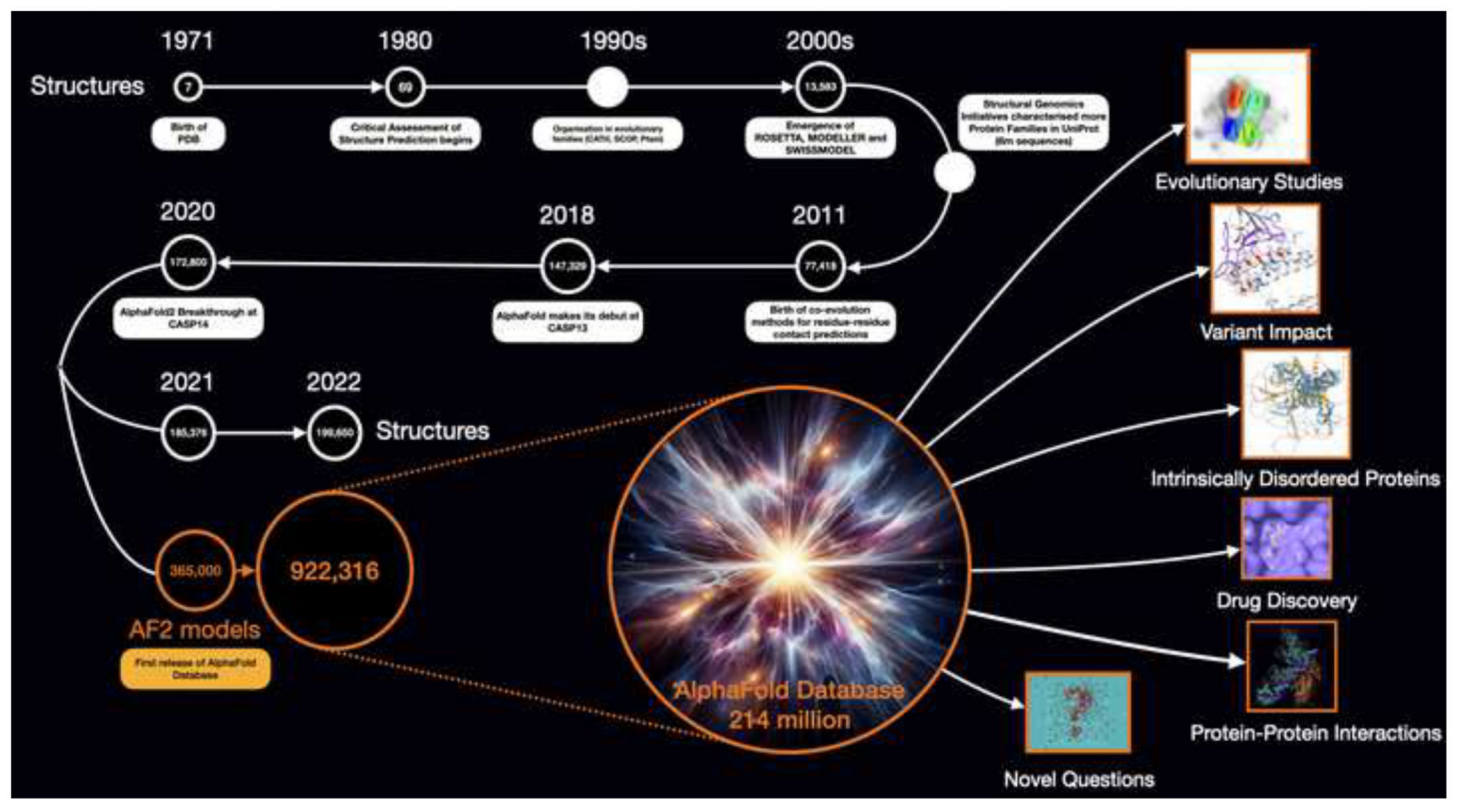
Milestones in the analyses of the protein universe. Growth of the Protein Data Bank and AlphaFold Database are shown within a timeline with major breakthroughs in software for protein structure prediction.
